# Study on the Spectrum-Effect Correlation of Anti-Inflammatory Active Extract of *Sauropus spatulifolius* Beille

**DOI:** 10.1155/2022/5646546

**Published:** 2022-05-24

**Authors:** Qin Qiu, Lujuan Jiang, Chunying Huang, Lifeng Yu, Dandan Zhen, Meifang Ye, Yuanyuan Liu, Junhao Shi, Xiaofang Liu, Baojun Gu, Hanshen Zhen

**Affiliations:** ^1^Guangxi University of Chinese Medical, Nanning 530001, China; ^2^Guangxi Superior Chinese Patent Medicine and National Medicine Development Engineering Technology Research Center, Nanning 530001, China; ^3^Traditional Chinese Medicine Hospital of YuLin, Yulin, China; ^4^Affiliated Hospital of Guilin Medical University, Guilin, China

## Abstract

*Sauropus spatulifolius* Beille (*S. spatulifolius*) is a commonly used medicine of the Bourau and Yao nationalities. However, the composition of *S. spatulifolius* is complex, and simple chemical fingerprints cannot accurately evaluate the relationship between its composition and efficacy. In this study, high-performance liquid chromatography (HPLC) method was used to establish the fingerprint of the ethyl acetate extract of *S. spatulifolius*. Based on the evaluation of the similarity of chromatographic fingerprints of traditional Chinese medicine, combined with cluster analysis and principal component analysis (PCA), the common peaks of fingerprints were evaluated. The anti-inflammatory effect data were extracted through the dimethylbenzene-induced ear-swelling model in mice. The gray relational analysis (GRA) combined with partial least squares regression (PLSR) was used to study the spectrum-effect correlation of *S. spatulifolius*. As a result, the HPLC fingerprint of the ethyl acetate extract of *S. spatulifolius* was established, and 18 common peaks were identified. Except for S6, the other similarities are all above 0.915. The reference substance control method was used to identify two absorption peaks, namely, protocatechuic acid and caffeic acid. The cluster analysis results showed that 10 samples from different origins were grouped into four categories, which was consistent with the PCA results. Ethyl acetate extract of 10 batches of *S. spatulifolius* could significantly inhibit the ear swelling of mice (*P* < 0.01). Through GRA, the order of the contribution of each chemical component to the anti-inflammatory efficacy was obtained. The results of PLSR showed that the VIP values of peaks 3, 4, and 12 were greater than 1 and were positively correlated with anti-inflammatory activity. In this study, the HPLC fingerprint of the ethyl acetate extract of *S. spatulifolius* was established. Through the study of the spectrum-effect correlation, the anti-inflammatory active substance of the ethyl acetate extract of *S. spatulifolius* was obtained. The anti-inflammatory effect of *S. spatulifolius* was the result of the joint action of multiple ingredients. This research helps to quickly and accurately discover the active ingredient groups of traditional Chinese medicine and provides new ideas and methods for studying the effective substances of traditional Chinese medicine.

## 1. Introduction

Medicinal plants are found nearly throughout the world and have been used for therapeutic, religious, cosmetic, nutritional, and beautification purposes since ancient times, and the humanity of all civilizations and culture are familiar with their usages. They have been recognized for their human health benefits also. They have high content of nonnutritive, nutritive, and bioactive compounds, such as flavonoids, phenolics, anthocyanins, and phenolic acids, which have potent antioxidant, anticancer, and antimutagenic effects [1–4]. *Sauropus spatulifolius* Beille (*S. spatulifolius*) is a plant of the Euphorbiaceae. It can be picked all year round or collected in summer and autumn. It is better to pick the green old leaves and dry them in the sun [5]. *S. spatulifolius* is a small evergreen shrub that mainly grows in valleys, mountain slopes, and moist and fertile jungles, distributed in Guangdong, Guangxi, Fujian, Hainan, and other regions [6]. The whole plant of *S. spatulifolius* can be used as medicine. In traditional medicine, the mature leaves of *S. spatulifolius* are generally used as medicine [7]. In clinical practice, *S. spatulifolius* is mainly used for clearing away heat and moisturizing lungs, resolving phlegm and relieving cough, improving pharynx, moistening intestines, and laxatives. *S. spatulifolius* mainly treats diseases such as lung heat, cough, sputum, constipation, dry mouth, sore throat, bronchitis, and bronchial asthma [5–8]. Modern pharmacological studies have shown that *S. spatulifolius* has anti-inflammatory and analgesic [9], antiallergic [10], antioxidation [11–13], antibacterial, antitussive, and expectorant effects [14,15].

Traditional Chinese medicine composition is complex, and its effect is usually the result of many components, many targets, and many ways. Traditional Chinese medicine fingerprint is a comprehensive and quantifiable identification method, which plays an important role in the identification and quality control of traditional Chinese medicine [16,17]. Although fingerprint is a widely accepted method for quality evaluation of traditional Chinese medicine in China, the simple chemical fingerprint cannot accurately evaluate the relationship between the composition and efficacy of traditional Chinese medicine. Therefore, it is necessary to change the traditional evaluation method based on chemical composition into one based on biological effect. The spectrum-effect correlation is a method to study the active components and quality control of traditional Chinese medicine. Through various mathematical model methods, the relation between fingerprint characteristic peak and drug effect is established to find out the substance that really has drug effect. This method makes up for the deficiency of separating the chemical composition from the medicinal effect. It provides a reliable method to elucidate the material basis of traditional Chinese medicine [18–20]. At present, the spectrum-effect relationship has been widely used in traditional Chinese medicine research, including the study of single medicinal substance of traditional Chinese medicine [21], medicinal substance of compound preparation [22, 23], compatibility of components [24], processing mechanism [25], and process optimization [26], providing more scientific basis for quality control of traditional Chinese medicine. It has made important contributions to its internationalization and globalization spread.

*S. spatulifolius* is a commonly used folk Zhuang Yao medicine. Now, it was included in Chinese pharmacopoeia [27]. Studies on quality control are mainly conducted by means of microscopic identification, spectral and chromatographic identification, heavy metal and pesticide inspection, and content determination, etc [28–33]. The fingerprint and spectrum-effect correlation of ethyl acetate extract of *S. spatulifolius* were not publicly reported. Therefore, high-performance liquid chromatography (HPLC) was used to establish the fingerprint effect of ethyl acetate extract of *S. spatulifolius,* and anti-inflammatory activity was used as a point of penetration. The correlation between common peaks and anti-inflammatory activity was studied by gray relational analysis (GRA) and partial least squares regression (PLSR) to further reveal the material basis of the anti-inflammatory effect of ethyl acetate extract of *S. spatulifoliu*s, in order to provide methods and basis for further research and quality control of *S. spatulifoliu*s.

## 2. Materials and Methods

### 2.1. Samples, Reagents, and Animals

The 10 batches of *S. spatulifolius* used in the experiment were all collected from Guangxi and Guangdong provinces. The plant was identified by Ma Life Deputy Chief Pharmacist (Deputy Chief Pharmacist of Guangxi Yixin Pharmaceutical Co., Ltd.; see Table 1 for details). Voucher specimens were preserved in the Department of Drug Analysis, Guangxi University of Chinese Medicine (No. 20180501). Methanol and acetonitrile were of chromatographic grade, purchased from Thermo Fisher Scientific Co., Ltd. (Massachusetts, USA); water was ultrapure; Tween-80 was of chemical grade, purchased from Tianjin Fuyu Fine Chemical Co., Ltd. (Tianjin, China, No: 20180928). Edible alcohol was of ordinary grade, purchased from Guangxi Haiying Alcohol Co., Ltd. (Nanning, China, No: 2016060701). Petroleum ether, ethyl acetate, n-butanol, glacial acetic acid, phosphoric acid, and xylene were all of analytical grades. Caffeic acid reference substance was purchased from China Institute for the Control of Pharmaceutical and Biological Products (Beijing, China, No: 110885-201703). The protocatechuic acid reference substance was purchased from China Institute for the Control of Pharmaceutical and Biological Products (Beijing, China, No: 111530-200303). Dexamethasone acetate tablets were purchased from Zhejiang Xianju Pharmaceutical Co., Ltd. (Zhejiang, China, No.: 170512). Sodium chloride injection was purchased from Huiyinbi Group Jiangxi East Asia Pharmaceutical Co., Ltd. (Jiangxi, China, No.: 2018112215).

KM mice (SPF grade) were supplied by Hunan Slike Jingda Laboratory Animal Co., Ltd. (approval No : SCXK (Xiang) 2016-0002. Changsha, Hunan, China). The Animal Ethics Committee of Guangxi University of Chinese Medicine approved all animal protocols. The animal experiments were carried out according to the Guide for the Care and Use of Laboratory Animals.

### 2.2. Apparatus and Conditions

The following apparatus and conditions were used: Waters e2695 high-performance liquid chromatograph (Waters 2489UV detector; Waters Corporation, USA); Thermo Syncronis C18 column (4.6 mm × 250 mm, 5 *μ*m); column temperature, 25°C; and detection wavelength, 260 nm. The flow rate was 0.8 mL·min^−1^. The injection volume was 10 *μ*L. The mobile phase was a mixture of methanol (A) and 0.1% glacial acetic acid (C). The gradient elution: 0–30 min, 15–18% A; 30–55 min, 18–30% A; 55–75 min, 30–35% A; 75–90 min, 35–38% A; 90–95 min, 38–15% A.

### 2.3. Preparation of Sample Solution

Preparation of extracts from different polar parts of *S. spatulifolius* is as follows: take 750 g dried leaves of *S. spatulifolius* and cut into fragments; add seven times 75% ethanol to soak for 4 d. Percolated extraction until the color of the extract becomes pale, the ethanol solvent was recovered, and the concentrated extract liquid was evaporated to dryness in a water bath to obtain a 75% ethanol total extract. The total extract of 75% ethanol was dissolved with appropriate amount of distilled water and petroleum ether, ethyl acetate, and n-butanol solvents used in the extraction were recovered. The concentrated extract liquid was evaporated to dryness by water bath kettle and stored in the desiccator for subsequent experiments.

Samples solution for the HPLC fingerprinting analysis is prepared as follows: 0.1 g of ethyl acetate extract of *S. spatulifolius* was (equivalent to 4.77 g of crude drugs) precisely weighed; it was dissolved by ultrasound and transferred to a 2 ml volumetric flask. Methanol was added to the volumetric flask to scale and centrifuged 10 min at a speed of 13,000 r·min^−1^. The supernatant was taken as the sample solution for HPLC analysis.

Mixed reference solution: appropriate amounts of caffeic acid and protocatechuic acid were precisely weighed and placed in 5 ml volumetric flasks and added methanol to scale, and then served as the reference stock solution of caffeic acid and protocatechuic acid. Appropriate amounts of caffeic acid reference stock solution and protocatechuic acid reference stock solution were precisely removed and placed in a volumetric flask of 2 mL, and added methanol to scale. Centrifuged 10 min at a speed of 13,000 r·min^−1^. The supernatant was taken as the mixed reference solution (containing 159.96 *μ*g mL^−1^ caffeic acid and 99.43 *μ*g mL^−1^ protocatechuic acid).

Sample solutions for animal experiments administration: take 10 batches of ethyl acetate extract from *S. spatulifolius* and add 0.1% Tween solution to prepare a solution equivalent to 3.4 g/kg of crude drug, store in the refrigerator at −5°C to be used in animal experiments.

### 2.4. HPLC Fingerprint Analysis Method Validation

The HPLC fingerprint analysis method was validated using parameters such as precision, repeatability, and stability of sample. The precision was determined by analyzing one sample six times continuously. The repeatability was carried out using six independent sample solutions. The stability of sample was determined by analyzing one sample at 0 h, 2 h, 4 h, 8 h, 12 h, and 24 h, respectively.

### 2.5. Animal Experiments

KM male mice (SPF grade) weighing 18–22 g was used for the experiment. They were housed in cages; on average, a mice was fed about 3 g.d^−1^ with a constant temperature (23 ± 1°C) and with a 12 h light/dark cycle. All animals underwent an adaptation period of 3 days. Male mice were randomly divided into 12 groups with eight mouse in each. Group 1 was the model group (mice were given normal saline at the same volume), group 2 was the positive group (mice were given 3 mg/kg dexamethasone acetate solution at the same volume), groups 3–12 were the medication administration groups (mouse were given 3.4 g/kg ethyl acetate extracts of *S. spatulifolius* [S1–S10]). The dosage volume of each group was 0.2 ml /10 g. Meanwhile, all treatment groups were given intragastric administration once a day for 10 days. About 45 min after the last administration, 10 microliter of dimethylbenzene was applied to the right ear of each group of mice, and the left ear was used for control. After 15 min of dimethylbenzene application, the mice were sacrificed by cervical dislocation, both ears of the mice were cut off and placed symmetrically, and round ear pieces were formed with an 8 mm punch and precisely weighed. The difference in weight between the ear pieces indicated the degree of swelling and calculated swelling inhibition rate. The calculation formula is as follows:

(1) $$\begin{array}{c} \text{degree of swelling}=\text{right ear piece weight}-\text{left ear piece weight,} \end{array}$$

(2) $$\begin{array}{c} \text{swelling inhibition rate}=\frac{\left(\text{average degree of swelling in the model group}-\text{average degree of swelling in the drug group}\right)}{\text{average degree of swelling in the model group}}\times 100\%. \end{array}$$

### 2.6. Data Handling

“Chinese traditional medicine chromatographic fingerprint similarity evaluation system (2012, 1 Edition)” was used to evaluate 10 batches of AIA data of ethyl acetate extract of *S. spatulifolius*. IBM SPSS Statistics 22.0 software was used to perform systematic clustering analysis on the original peak areas of 18 common peaks in the fingerprints of ethyl acetate extract of 10 batches of *S. spatulifolius*. Cluster analysis was performed using between-groups linkage, and the distance between samples was calculated using squared Euclidean distance. IBM SPSS Statistics 22.0 software was used to carry out principal component analysis (PCA) on the peak areas of 18 common peaks in the ethyl acetate extract of 10 batches of *S. spatulifolius*, and the eigenvalues, variance contribution rate, and cumulative contribution rate of each component were obtained.

IBM SPSS Statistics 22.0 software was used for statistical analysis of animal experimental data. One-way analysis of variance was used for analysis, and *t* test was used for comparison between groups. The degree of swelling was expressed as ‾*x* ± *s*. The difference between the model group (normal saline group) and each administration group was compared. *P* < 0.05 indicated that the difference was statistically significant, and *P* > 0.05 indicated that the difference was not statistically significant.

The inhibition rate of dimethylbenzene-induced ear swelling in mice by extracts of ethyl acetate extract of *S. spatulifolius* from different origins was used as the parent sequence, and the peak areas of 18 common peaks in the HPLC fingerprint were used as the subsequence. For calculation methods and steps, refer to [34, 35] for GRA. The peak area of the common peaks in the HPLC fingerprint was taken as the independent variable (X), the peak areas of the No. 1 peak to the No. 18 peak were recorded as x1–x18 in turn, and the inhibition rate of the mice ear swelling caused by dimethylbenzene was the dependent variable (Y) . The data were imported into SIMCA-P software for PLSR.

## 3. Results

### 3.1. Anti-Inflammatory Effect

In this study, dimethylbenzene induced ear swelling in mice was used to evaluate extracts of ethyl acetate extract of *S. spatulifolius* anti-inflammatory activity. Due to the large number of mice, 10 batches of ethyl acetate extracts from *S. spatulifolius* were divided into two experiments. The previous study by our research group found that the high-dose ethyl acetate extract group (3.40 g/kg) had the best inhibitory effect on mice ear swelling. Therefore, the most effective dose of 3.40 g/kg was selected as the administration dose for mice. The results showed (Table 2 and Table 3) that compared with the model group, the ethyl acetate extract group of the S1–S10 samples significantly reduced the degree of mice ear swelling (^*∗*^*P*  < 0.05; ^*∗∗*^*P* < 0.01), and the ethyl acetate extracts of *S. spatulifolius* from different origins could inhibit the ear swelling of mice.

### 3.2. Fingerprint Analysis

In the process of establishing the fingerprint in this experiment, the chromatographic conditions were investigated, and it was found that when methanol –0.1% glacial acetic acid solution was used as the mobile phase, the detection wavelength was 260 nm, the column temperature was 25°C, and the flow rate was 0.8 mL/min, the separation effect and peak shape of each chromatographic peak were good, the baseline was stable, and the number of peaks was large. The method validation results of fingerprint established showed that the relative standard deviations of relative retention times for each chromatographic peak were less than 1%, and the relative standard deviations of relative peak areas were less than 3.02%. It was indicated that the precision of instrument and the repeatability of extraction method was good, and the sample was stable in 24 h.

**Figure 1:**
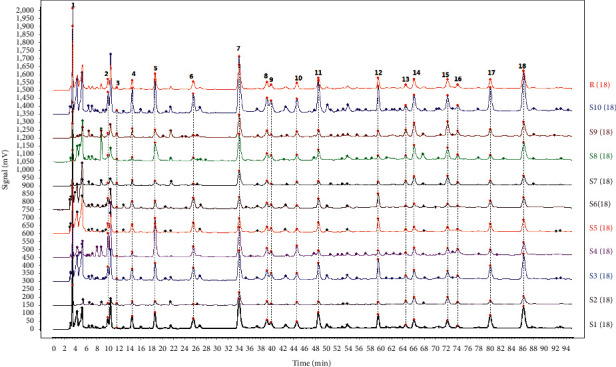
The overlay chromatograms of 10 batches of ethyl acetate extracts of *S. spatulifolius*.

**Figure 2:**
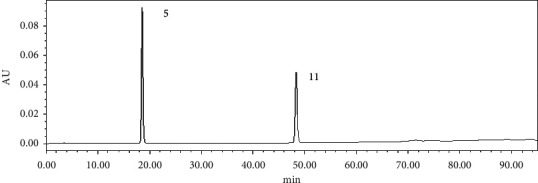
The chromatogram of the mixed reference substance.

**Figure 3:**
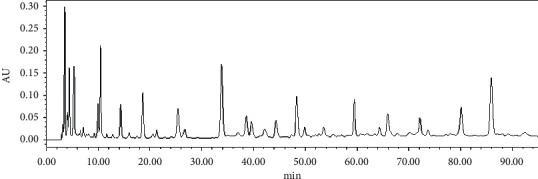
The chromatogram of ethyl acetate extract of *S. spatulifolius*.

Comparing the chromatograms of 10 batches of ethyl acetate extracts from *S. spatulifolius*, 18 common peaks were identified, of which the 5th peak was protocatechuic acid and the 11 th peak was caffeic acid. Protocatechuic acid has good resolution and peak shape and moderate retention time, so it was used as the reference peak. The overlay chromatograms of 10 batches of ethyl acetate extracts of *S. spatulifolius* is shown in Figure 1, the chromatogram of the mixed reference substance is shown in Figure 2, and the chromatogram of the ethyl acetate extract is shown in Figure 3. A total of 10 batches of ethyl acetate extracts from *S. spatulifolius* fingerprint similarity evaluation results are shown in Table 4. The results showed that, except for S6, which had a similarity of 0.879, the similarity of the other batches of samples were all greater than 0.9, indicating that the quality of *S. spatulifolius* in each production area was basically stable, and the similarity of individual production areas was low, which may be related to the growth environment, harvesting season, cultivation method, processing method, and other factors.

**Figure 4:**
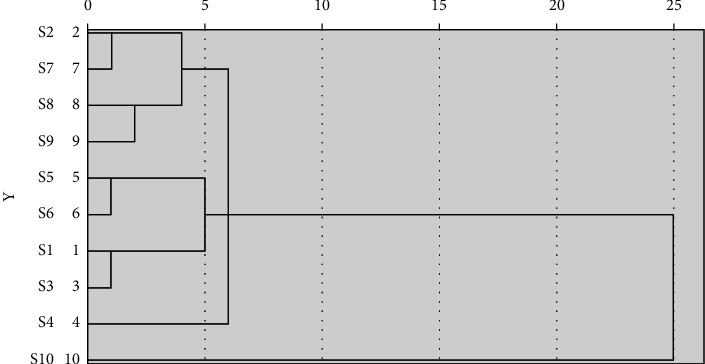
Cluster analysis results of fingerprints of 10 batches of ethyl acetate extracts from *S spatulifolius*.

The cluster analysis results are shown in Figure 4. The results showed that 10 batches of *S. spatulifolius* were grouped into four categories,the first category included S2, S7, S8, and S9; the second category included S1, S3, S5, and S6; the third category included S4; the fourth category included S10. The results showed that the sample classification was not affected by the origin, but may be related to the content of some chemical components.

**Figure 5:**
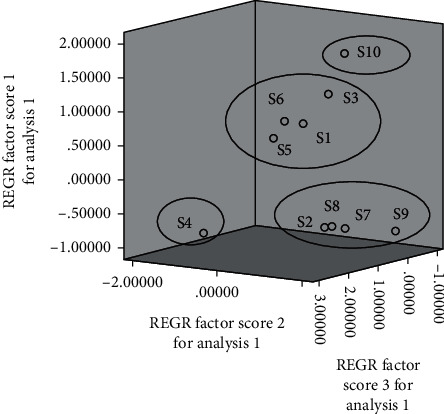
3D diagram of principal component score.

PCA was a widely used statistical method, which was used to simplify data and quickly realize the visual pattern recognition of pattern or relationships. The principal component analysis results of 10 batches of ethyl acetate extracts from *S. spatulifolius* showed that with the characteristic value was greater than 1 as the principle of principal component extraction stated, three principal components could be extracted, and the 3D diagram of the principal components was shown in Figure 5. As shown in Table 5, the cumulative variance contribution rate of the first three principal components was 94.776%, which could comprehensively reflect the basic characteristics of its chemical components. It indicated that these three principal components represented most of the information contained in the 18 common peaks of the ethyl acetate extract of *S. spatulifolius*. From the rotation component matrix (Table 6), it was speculated that the reason for the difference in the quality of various batches of *S. spatulifolius* was the result of the combined effect of various chemical components. The variance contribution rate of the first principal component was 60.814%, which was the most “informative” component, and its information mainly came from chromatographic peaks 1, 2, 4, 6–9, 11, 12, 17, and 18; The variance contribution rate of the second principal component was 20.249%, and the information mainly came from chromatographic peaks 3, 13–15; The variance contribution rate of the third principal component was 13.713%, and the information mainly came from the 2, 5, 10 and 16 chromatographic peaks.

### 3.3. Spectrum-Effect Correlation Analysis

GRA is a quantitative analysis method in gray system theory, which is used to measure the gray relational degree between the target and the factors. The greater the gray correlation degree between the two, the greater the correlation, and the smaller the gray correlation degree, the smaller the correlation [36]. In the establishment of the spectral-effect relationship, the GRA method mainly analyzes the correlation between the components and efficacy, but it cannot determine whether the characteristic peaks have a positive or negative effect on the efficacy. Compared with GRA, PLSR is a common technique and has been successfully applied to solve many practical problems, and this method is simple and accurate and can describe how each characteristic peaks contribute positively or negatively to the efficacy, and it gives the magnitude of the contribution, but the ability to analyze the correlation between composition and effect is poor. Therefore, the combined application of GRA and PLSR can draw on each other's strengths to more accurately to analyze the spectral effect correlation of traditional Chinese medicine [7–12].

**Figure 6:**
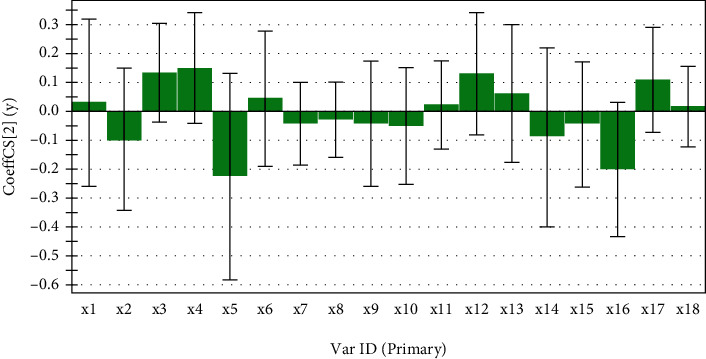
Partial regression coefficient plot of common peaks and anti-inflammatory efficacy values.

**Figure 7:**
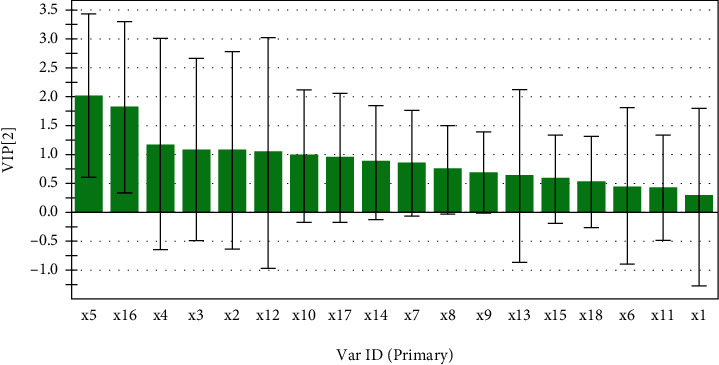
VIP contribution plot of common peak anti-inflammatory efficacy.

The GRA results were shown in Table 7. The correlation degree basically reached 0.8. Except for the No. 5 peak and No. 16 peak, the correlation degrees of the rest of the characteristic peaks were all greater than 0.8. It showed that the anti-inflammatory effect of ethyl acetate extract of *S. spatulifolius* was the result of the synergistic effect of multiple components. According to the degree of correlation, the order of contribution of each component to anti-inflammatory effect was determined as 8 > 13>14 > 7>2 > 15>18 > 9>10 > 1>6 > 12>4 > 17 = 11 > 3>5 > 16. PLSR obtained the partial regression coefficients (Figure 6) and VIP values (Figure 7) of 18 characteristic peaks (*X*) and inhibition rates (Y). Among the characteristic peaks of ethyl acetate extract of *S. spatulifolius*, nine peaks were positively correlated with the inflammation inhibition rate, and the remaining nine peaks were negatively correlated with the inflammation inhibition rate. The contribution of X to Y was determined according to the size of the VIP information. Generally speaking, the greater the VIP value, the greater the correlation between the variable and the drug efficacy. When VIP>1, this characteristic peak has a significant contribution to the inflammation inhibition rate. The results showed that the VIP values of peaks 5, 16, 4, 3, 2, and 12 were greater than 1. It showed that in the HPLC fingerprint of ethyl acetate extract of *S. spatulifolius*, its corresponding components played an important role in the inhibition rate of inflammation. The GRA and PLSR were combined to analyze, and the gray correlation degree>0.8, the partial least squares regression coefficient was positive, and the VIP value was greater than 1 for the chromatographic peak screening conditions. Finally, it was determined that the corresponding components of absorption peaks 3, 4, and 12 were the components in the ethyl acetate extract of *S. spatulifolius* that played an important role in the anti-inflammatory effect of mice.

## 4. Conclusions

In this study, the fingerprints of 10 batches of ethyl acetate extracts from *S. spatulifolius* were established, and 18 common peaks were identified. The evaluation results of the similarity of fingerprints showed that as excepted for S6, the other similarities were all above 0.915. Two peaks were identified by the reference method: protocatechuic acid absorption peak and caffeic acid absorption peak. The results of cluster analysis showed that samples from 10 different origins were clustered into four categories, which were consistent with the results of PCA. It showed that this method was feasible for analysis. In this study, GRA and PLSR were used to study the relationship between the common peaks of HPLC fingerprints of ethyl acetate extract of *S. spatulifolius* and the inhibition rate of inflammation. It was indicated that the corresponding components of No. 3, 4, and 12 absorption peaks in the ethyl acetate extract of *S. spatulifolius* were closely related components of the anti-inflammatory function of *S. spatulifolius*, and the spectrum-effect correlation between the ethyl acetate extract of *S. spatulifolius* and the anti-inflammatory activity was preliminarily expounded, which laid the foundation for the research on its pharmacodynamic substances. Therefore, chemical separation, LC-MS and other means could be used for further analysis to explore the substances represented by these three absorption peaks. The anti-inflammatory efficacy experiment was carried out on the three characteristic peak compounds separated and analyzed to further determine the anti-inflammatory efficacy of the compounds, which was of great significance for the determination of quality markers and quality control of *S. spatulifolius* medicinal materials.

## Figures and Tables

**Table 1:** Information of *S. spatulifolius* samples.

Numbers	Producing areas	Purchasing period
S1	Hepu , Beihai	2018.07
S2	Wuming, Nanning	2018.07
S3	Fucheng , Beihai	2018.08
S4	Cen xi, Wuzhou	2018.08
S5	Shatian, Beihai	2018.11
S6	Jinghai, Beihai	2018.11
S7	Nanning qingxiu district	2019.01
S8	Zhaoqing, Guangzhou	2019.01
S9	Hengxian, Nanning	2019.02
S10	Xinggang, Beihai	2019.02

**Table 2:** Experimental results of ear swelling induced by dimethylbenzene in mice (*n* = 8).

Groups	Dosage of administration (g/kg)	Swelling degree (mg) (‾*x* ± *s*)	Inhibition ratio (%)
Model group	—	4.9 ± 1.0	—
Positive group	0.003	1.6 ± 0.7^*∗∗*^	67.35
Wuming, Nanning	3.4	2.4 ± 1.0^*∗∗*^	51.02
Cen xi, wuzhou	3.4	2.7 ± 1.6^*∗∗*^	44.90
Nanning qingxiu district	3.4	2.6 ± 1.0^*∗∗*^	46.94
Zhaoqing, Guangzhou	3.4	2.8 ± 1.0^*∗∗*^	42.86
Nanning hengxian	3.4	2.5 ± 1.1^*∗∗*^	48.95

*Note.* Compared with model group^*∗*^*P*  < 0.05; ^*∗∗*^*P* < 0.01.

**Table 3:** Experimental results of ear swelling induced by dimethylbenzene in mice (*n* = 8).

Groups	Dosage of administration (g/kg)	Swelling degree (mg) (‾*x* ± *s*)	Inhibition ratio (%)
Model group	—	8.7 ± 1.7	—
Positive group	0.003	1.6 ± 0.6^*∗∗*^	81.61%
Hepu , Beihai	3.4	4.5 ± 1.7^*∗∗*^	48.28%
Fucheng , Beihai	3.4	4.3 ± 1.7^*∗∗*^	50.57%
Shatian, Beihai	3.4	4.7 ± 1.6^*∗∗*^	45.98%
Jinghai, Beihai	3.4	4.5 ± 1.7^*∗∗*^	48.28%
Xinggang, Beihai	3.4	4.6 ± 1.4^*∗∗*^	47.13%

*Note.* Compared with model group^*∗*^*P*  < 0.05; ^*∗∗*^*P* < 0.01.

**Table 4:** 10 batches of ethyl acetate extracts from *S. spatulifolius* similarity evaluation results.

Numbers	Similarity
S1	0.991
S2	0.951
S3	0.985
S4	0.915
S5	0.934
S6	0.879
S7	0.950
S8	0.952
S9	0.951
S10	0.990

**Table 5:** Eigenvalues and contribution rates of principal components.

Principal components	Initial eigenvalues		
	Total	Variance contribution rate (%)	Cumulative contribution rate (%)
1	10.946	60.814	60.814
2	3.645	20.249	81.063
3	2.468	13.713	94.776

**Table 6:** Rotation component matrix.

Peaks	Principal components			Peaks	Principal components		
	1	2	3		1	2	3
1	0.918	−0.206	0.101	10	0.552	0.362	0.733
2	0.624	−0.167	0.736	11	0.902	0.346	0.196
3	−0.180	0.720	−0.406	12	0.989	0.035	−0.042
4	0.980	0.134	−0.024	13	0.098	0.978	0.097
5	0.230	0.166	0.940	14	0.229	0.902	0.283
6	0.940	−0.187	0.239	15	0.116	0.955	0.227
7	0.611	0.527	0.575	16	−0.212	0.066	0.908
8	0.694	0.489	0.527	17	0.894	0.353	0.194
9	0.818	0.318	0.444	18	0.778	0.529	0.285

**Table 7:** Results of GRA.

Peaks	Correlation
1	0.8238
2	0.8392
3	0.8031
4	0.8129
5	0.7796
6	0.8204
7	0.8497
8	0.8747
9	0.8324
10	0.8318
11	0.8090
12	0.8130
13	0.8621
14	0.8581
15	0.8385
16	0.7416
17	0.8090
18	0.8352

## Data Availability

The data used to support the findings of this study are included within the article.
